# The relationship between folding and activity in UreG, an intrinsically disordered enzyme

**DOI:** 10.1038/s41598-017-06330-9

**Published:** 2017-07-20

**Authors:** Marta Palombo, Alessio Bonucci, Emilien Etienne, Stefano Ciurli, Vladimir N. Uversky, Bruno Guigliarelli, Valérie Belle, Elisabetta Mileo, Barbara Zambelli

**Affiliations:** 1 Laboratory of Bioinorganic Chemistry, Department of Pharmacy and Biotechnology, University of Bologna, Viale G. Fanin 40, Bologna, 40127 Italy; 2 Aix-Marseille Univ, CNRS, IMM (FR 3479), BIP (UMR 7281), 31 chemin Joseph Aiguier, Marseille, 13402 France; 3 Department of Molecular Medicine, University of South Florida, 12901 Bruce B. Downs Blvd., Tampa, MDC07 USA

## Abstract

A growing body of literature on intrinsically disordered proteins (IDPs) led scientists to rethink the structure-function paradigm of protein folding. Enzymes are often considered an exception to the rule of intrinsic disorder (ID), believed to require a unique structure for catalysis. However, recent studies revealed the presence of disorder in several functional native enzymes. In the present work, we address the importance of dynamics for catalysis, by investigating the relationship between folding and activity in *Sporosarcina pasteurii* UreG (*Sp*UreG), a P-loop GTPase and the first discovered native ID enzyme, involved in the maturation of the nickel-containing urease. The effect of denaturants and osmolytes on protein structure and activity was analyzed using circular dichroism (CD), Site-Directed Spin Labeling (SDSL) coupled to EPR spectroscopy, and enzymatic assays. Our data show that *Sp*UreG needs a “flexibility window” to be catalytically competent, with both too low and too high mobility being detrimental for its activity.

## Introduction

The lack of tertiary structure in functional proteins is an exciting discovery of the last couple of decades^1, 2^. The number of known intrinsically disordered proteins (IDPs) is constantly increasing, involving a plethora of functions, especially associated to regulatory and signaling functions. Enzymes, on the other hand, are traditionally viewed as an exception to the rule, through the lens of the rigid “lock-and-key” model. This idea, amended by the observation that enzymes may shift between diverse conformational states upon substrate binding and conversion^3^, is based on the belief that, if some protein flexibility is necessary for enzymes upon catalysis, major disorder is unfavorable for catalytic efficiency^4^. This theory is now challenged by the discovery of activity in some disordered enzymes, either natural or engineered^5, 6^. Indeed, several recent studies, addressing the role of protein dynamics in enzymes^4, 7, 8^, led to the progressive shift from a purely structure-centric idea of catalysis, in which enzymes should bind the substrate in a rigid pre-organized fashion, towards an ensemble-weighted allosteric view of enzymatic functions^9^. In particular, a recent study identified nearly 100 enzymes experimentally proven to contain disorder, and, using disorder predictions, concluded that flexibility occurs in enzymes with the same rate as in non-catalytic proteins^6^. The recurrence of ID in enzymes pinpoints its functional role for catalytic activity. So, why would enzymes require ID? In several instances, protein flexibility emerged as an essential tool for modulating enzyme specificity and self-regulation. In particular, disorder can control enzyme activity in two distinctive ways: with an internal mechanism (involving catalysis)^8, 10–12^, or with an external process (involving the interactions with regulators and/or via post-translational modifications)^13–16^.

**Figure 1:**
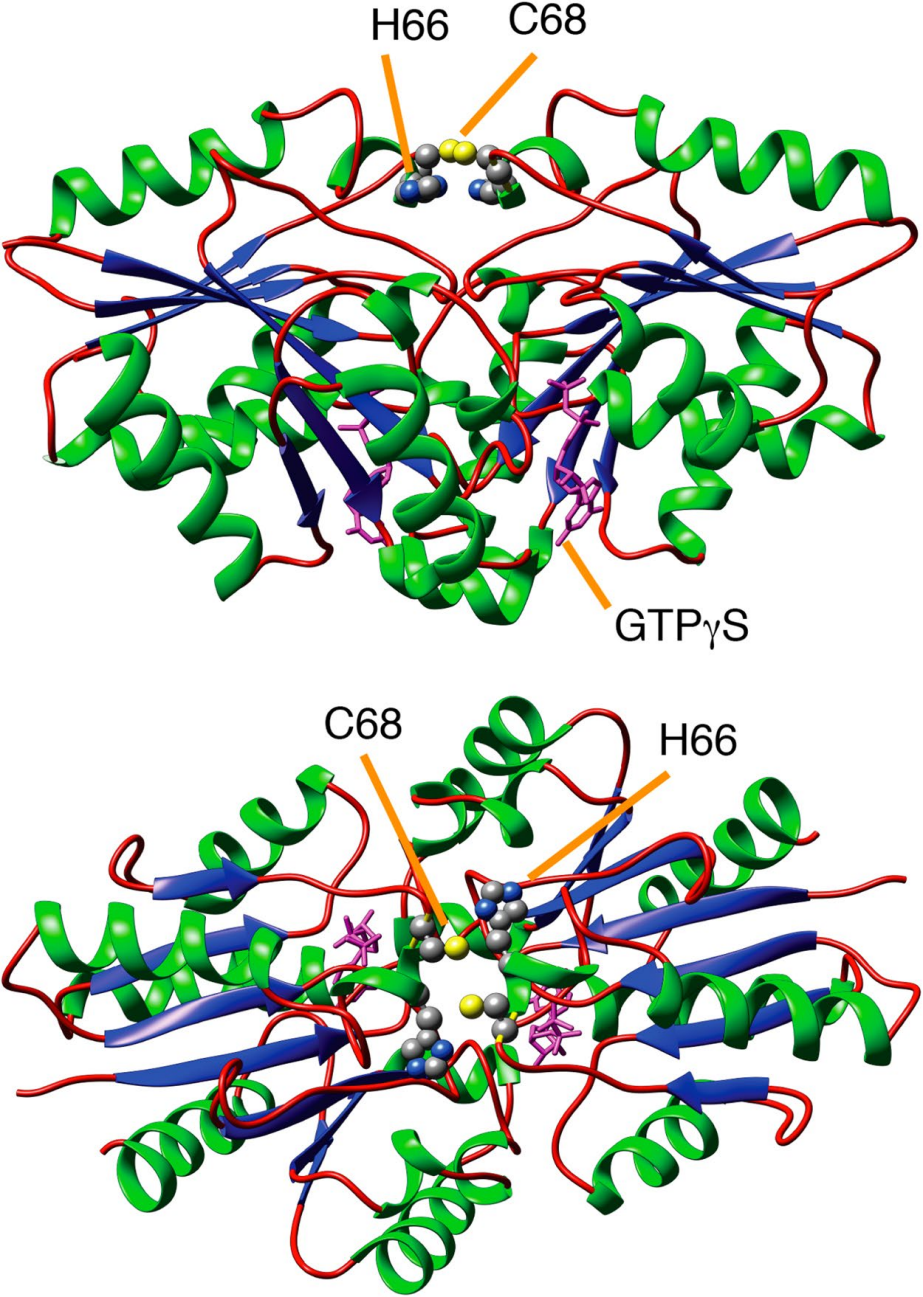
Cartoon representation of the ﻿previously reported﻿ model structure of *Sp*UreG in the fully folded functional state^25^. Metal binding residues His^66^ and Cys^68^ are indicated, as well as the GTPγS substrate analogue.

UreG is a GTP hydrolase that assists nickel delivery into nickel-dependent urease, a pathogenic factor for several bacteria and fungi. This GTPase functions in complex with three accessory proteins, namely UreF, UreD and UreE, that cooperate for urease activation forming a multimeric molecular chaperone^17^. We previously demonstrated that isolated UreG proteins from different domains of life exist in a conformational ensemble under native conditions^18–22^. In particular, UreG shows a significant content of secondary structure, as revealed by circular dichroism (CD), and a minor amount of tertiary structure, deduced both from intrinsic Trp fluorescence and from its ^1^H-^15^N HSQC NMR spectrum, showing broad signals with limited spread in ^1^H dimension, which indicate a backbone mobility in the intermediate exchange regime^23^. Some UreG proteins feature a moderate GTPase activity under native disordered conditions, and a variable degree of structural content in different orthologues, suggesting that the protein functioning *in vivo* passes through a *disorder-to-order* transition^18–22^. Indeed, the crystal structure of a well-folded dimeric conformer of UreG from *Helicobacter pylori* (*Hp*UreG) was reported in a complex with the urease accessory proteins UreF and UreD^24^. This suggests that the fully active structure of UreG *in vivo* can be stabilized only in a functional urease activation complex. Hence, disorder could be a way to regulate GTPase activity, avoiding unwanted GTP hydrolysis and consequently energy waste. A homology model of the dimeric fully folded state of UreG from *Sporosarcina pasteurii* (*Sp*UreG) was reported using *Hp*UreG crystal structure as a template (Fig. 1)^25^.

In the present study, we address the relationship between the structural plasticity and the enzymatic activity in *Sp*UreG, by perturbing the protein conformational landscape using denaturants and osmolytes and investigating its structure and function with circular dichroism (CD), Site-Directed Spin Labeling (SDSL) coupled to EPR spectroscopy, and enzymatic assays.

## Results and Discussion

### SDSL-EPR of native SpUreG shows a heterogeneous conformational ensemble

**Figure 2:**
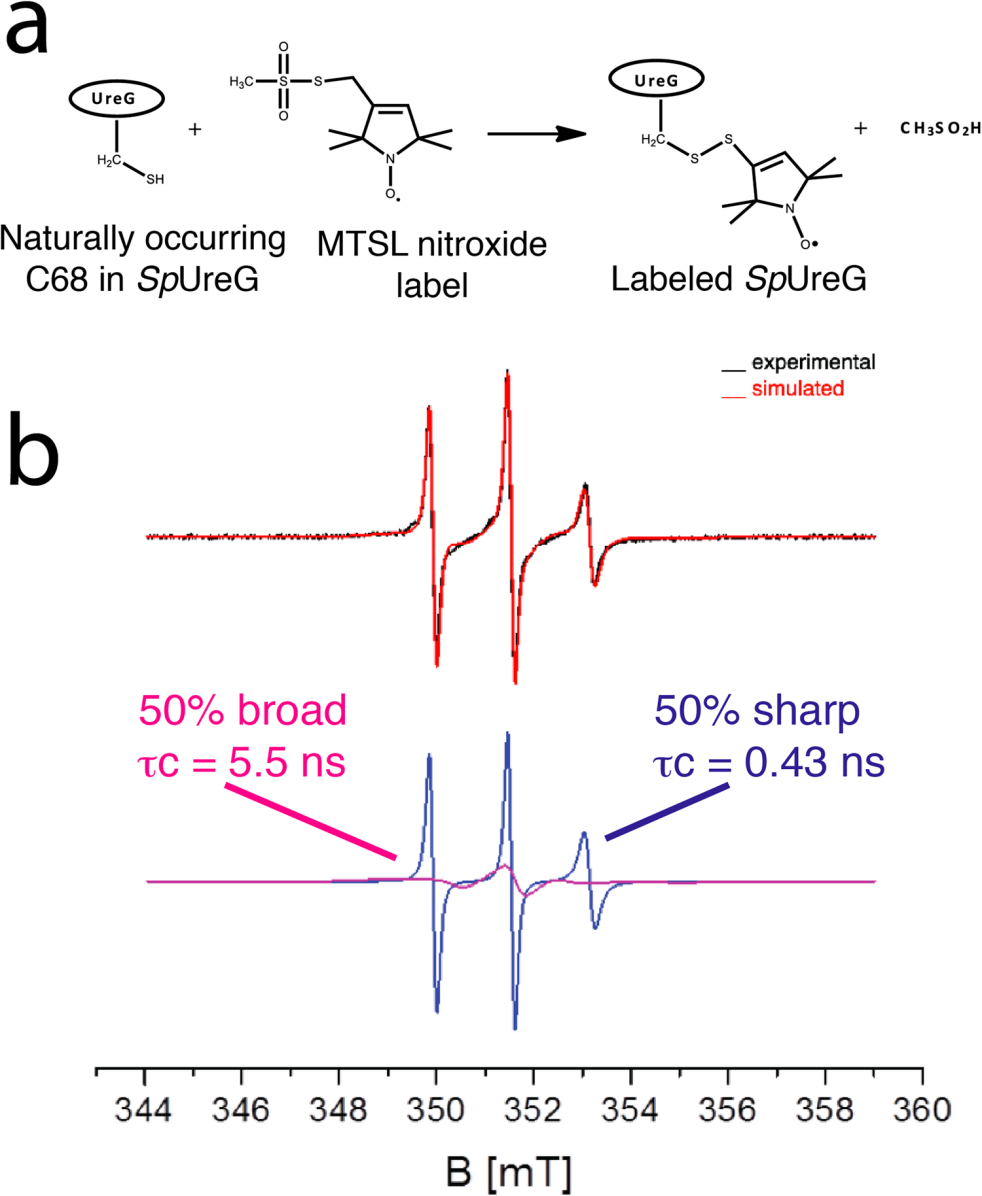
Folding state of *Sp*UreG. (**a**) Schematic representation of the labeling reaction of Cys^68^ with MTSL spin label. (**b**) *Top panel*: SDSL-EPR spectrum of *Sp*UreG-MTSL under native conditions in 20 mM TrisHCl pH 8, 150 mM NaCl (black) and the corresponding spectrum (red) simulated using SimLabel program^32^. *Bottom panel*: Decomposition of the simulated spectrum into one sharp and one broad component. The corresponding correlation time (*τ*_*c*_) is indicated, together with the relative proportions of different components.

To explore the conformational landscape of isolated *Sp*UreG, we applied Site-Directed Spin Labeling (SDSL) combined with Electron Paramagnetic Resonance (EPR) spectroscopy. In SDSL, a nitroxide label is specifically grafted to a cysteine residue (Fig. 2a) and is monitored by EPR. The shape of the continuous wave EPR spectrum reflects the mobility of the nitroxide, which acts as a sensitive reporter of the local environment and motion of the protein^26–28^. In particular, when the mobility of nitroxide varies in the ns-μs time scale, changes in the line-shape of the EPR spectrum are observed: higher degree of flexibility results in narrow line widths (the so-called *sharp* signal), while a decreased mobility is reflected by broader line widths (the so-called *broad* signal). The sensitivity to local dynamics has made SDSL-EPR a successful tool to investigate protein folding in IDP systems, especially to monitor *disorder-to-order* transitions^29, 30^.

We labeled *Sp*UreG with the MTSL spin label (S-(1-oxyl-2,2,5,5-tetramethyl-2,5-dihydro-1H-pyrrol-3-yl)methyl methanesulfonothioate) at the unique naturally occurring Cys^68^ (*Sp*UreG-MTSL) (Figs 1, 2a and Supplementary Figure 1). This *Sp*UreG modification with MTSL nitroxide (ca. 7 Å) does not affect the protein structure and activity, as verified comparing CD and activity assays on the labeled and non-labeled (native) protein (Supplementary Figure 2a and b). The resulting EPR spectrum exhibits a line-shape typical of the presence of multiple motional components (Fig. 2b). Indeed, simulation of the EPR spectrum with the SimLabel software (a GUI of EasySpin)^31, 32^, which provides a quantitative interpretation of the protein dynamics in terms of the rotational correlation time (*τ*_*c*_), reveals the presence of two major conformations in equal abundance, characterized by a *τ*_*c*_ = 0.43 ns for the narrower signal and *τ*_*c*_ = 5.5 ns for the broader signal (Supplementary Table 1-SI and Fig. 2b). The former is indicative of a high mobility of the spin label, consistent with a high flexibility and lack of secondary and/or tertiary structure in the region surrounding the label. A similar correlation time was found for other IDPs, such as the MTSL-labeled C-terminal domain of the measles virus nucleoprotein^29, 30^, for which we recently re-evaluated the EPR spectrum using SimLabel (see Supplementary Figure 3). On the other hand, the broad component of the spectrum is indicative of a protein conformer displaying a ten-fold decreased mobility. The composite EPR spectrum supports the idea that two conformational states of *Sp*UreG coexist in solution. A similar result was obtained by mass spectroscopy of native *Sp*UreG, which revealed the presence of multiple conformers with different degrees of folding, ranging from fully disordered to globular^33^.

In order to exclude that the heterogeneous behavior of *Sp*UreG in solution is caused by the occurrence of a monomer-dimer equilibrium, known to feature a dimerization constant of 45 µM^33^, we recorded the EPR spectrum of *Sp*UreG-MTSL at a concentration close to the dimerization constant (62 µM) as well as at a concentration well above the dimerization constant (260 µM). Only minor changes in the EPR spectra are visible under the two conditions, proving that the equilibrium between the two conformational states is not influenced by the monomer/dimer equilibrium (see Supplementary Figure 4).

### Denaturants and osmolytes affect the SpUreG structure

**Figure 3:**
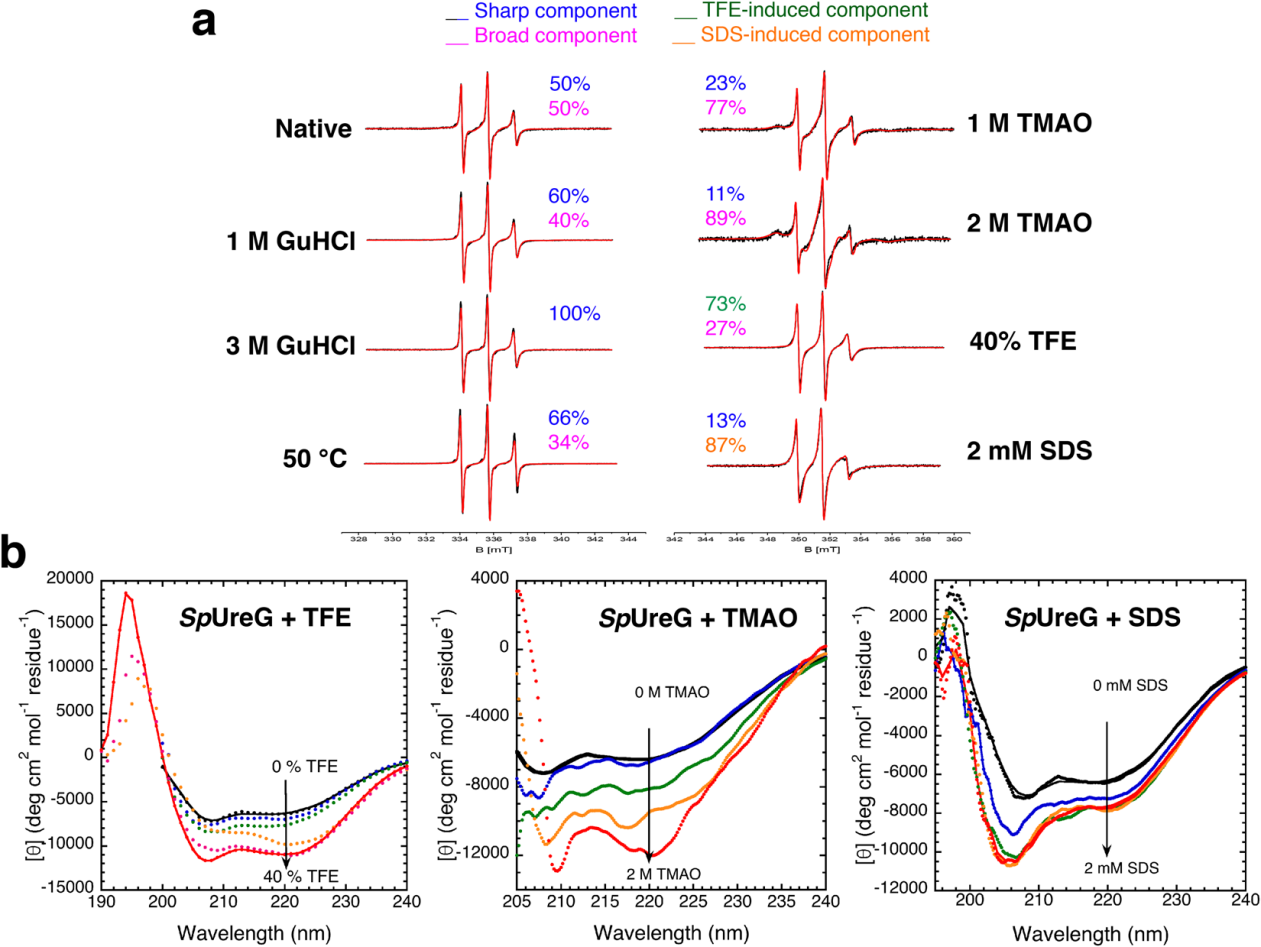
Effects of different additives and temperature on *Sp*UreG structural flexibility. (**a**) SDSL-EPR spectra of *Sp*UreG-MTSL (62 µM in 20 mM TrisHCl pH 8, 150 mM NaCl) at different temperatures and with various additives, as indicated. The spectra simulations (red) are superimposed to the EPR spectra (black). (**b**) Titration of *Sp*UreG (20 µM, in 20 mM TrisHCl pH 8, 150 mM NaCl, 1 mM TCEP) with different additives (TFE, TMAO, SDS), followed by CD. TFE concentrations: 0%, black; 5%, blue; 10%, green; 20%, orange; 30%, magenta; 40%, red. TMAO concentrations: 0 M, black; 0.4 M, blue; 1 M, green; 1.5 M, orange; 2 M, red. SDS concentrations: 0 mM, black; 0.5 mM, blue; 1 mM, green; 1.5 mM, orange; 2 mM, red.

A previous study using a combination of different techniques such as CD, intrinsic fluorescence and NMR, explored the chemical and thermal denaturation of *Sp*UreG and found that native *Sp*UreG exists as a dynamic ensemble of inter-converting, partially folded conformations that fully unfold at increasing concentrations of urea or guanidinium hydrochloride (GuHCl)^23^. Differently, temperature increase results in a non-cooperative transition leading to a non-native ensemble that retains significant secondary structural content but no tertiary structure and resembles a pre-molten globule conformation^23^. In the present work, these denatured ensembles were further characterized with SDSL-EPR: the addition of GuHCl selectively decreases the broad spectral features while increasing the sharp component of *Sp*UreG-MTSL, further confirming that the latter represents the unfolded state of *Sp*UreG. At 1 M GuHCl, a concentration sufficient to shift the conformational ensemble toward the pre-molten globule-like conformation^23^, the broad and sharp components are 40% and 60% respectively, while at 3 M GuHCl, in which the random coil-like state is prevalent^23^, no trace of the broad component is observed (Fig. 3a and Supplementary Table 1). Thermal unfolding, which also induces a change toward the pre-molten globule-like ensemble^23^, shows a sharp component of 66% at 50 °C and 100% at 80 °C (Fig. 3a and Supplementary Table 1). The temperature-dependent structural transition was partially reversible in SDSL-EPR (Supplementary Figure 5), as previously observed using CD and differential scanning calorimetry^23^. All together, these data confirm that both chemical denaturants and temperature affect the protein dynamics and thus the structural contacts in the labeled region.

It is known that intrinsically disordered proteins are, in general, very sensitive to changes in the solution conditions^34^, with most IDPs undergoing an induced folding transition upon interaction with chemical additives, such as osmolytes^34^. Therefore, in an attempt to study the structural and functional behavior of a folded state of *Sp*UreG, we perturbed its conformational landscape by adding folding-promoting agents, and measured their effects on secondary and tertiary structure using circular dichroism (CD) and SDSL-EPR. The far-UV circular dichroism (CD) spectrum of native *Sp*UreG, featuring negative deflections at 206 nm and at 220 nm, indicative of the presence of both α-helix and β-sheet content has been reported (Supplementary Figure 2a)^23^. The estimated content of secondary structure, derived from the CD spectrum using the Dichroweb server^35^, is 15% α-helices and 29% β-sheets^18^. Comparing these values with the secondary structure composition calculated using STRIDE^36^ for the molecular model of the protein in the fully folded state (39% α-helices and 17% β-sheets) (Fig. 1a)^25^, we deduced that under native conditions *Sp*UreG lacks about half of the α-helical content, while the β-strands, mainly situated in the protein core, are more largely conserved. Therefore, if a disorder-to-order transition occurs in *Sp*UreG upon functioning, the protein structural changes should involve mainly the α-helices^18^. On this basis, we tested the effect of trifluoroethanol (TFE), an additive known to enhance the helical content in IDPs with α-helical propensity^37^. TFE was titrated onto the protein solution and the CD spectra under different TFE concentrations were recorded (Fig. 3b). Overall, TFE produces an increase of the absolute value of ellipticity and a shift of the band at 220 nm toward 222 nm, indicating a significant expansion of the α-helical structure. Concentrations of TFE between 10% and 20% cause the most important change in ellipticity and the effect reaches the saturation at 40% of TFE. Under these conditions, a fit of the CD data estimated 51% α-helices and 20% β-sheets. The SDSL-EPR spectrum of *Sp*UreG-MTSL in the presence of 40% TFE, a concentration in which the structural effect of the additive onto the protein folding reaches the saturation from the CD spectra, indicates a significant decrease of the broad component from 50% to 27% and an increase of the correlation time of the sharp component (from *τ*_*c*_ = 0.43 ns to *τ*_*c*_ = 0.73 ns). The decrease in mobility of the latter conformation most likely reflects a gain of rigidity in the region of the label, possibly reflecting the TFE-induced secondary structure (Fig. 3a and Supplementary Table 1).

Trimethylamine N-oxide (TMAO), a naturally occurring osmolyte, is an additive able to force thermodynamically unstable proteins to fold and regain high functional activity^34^. Addition of a 1–2 M of TMAO to *Sp*UreG produces a major increase of protein ordered structure, as revealed by both CD, which was used for the titration of the conformational effect of the osmolyte, and SDSL-EPR. In particular, a more negative ellipticity is visible in the CD spectrum upon TMAO titration (Fig. 3b), and although a proper estimate of secondary structure composition using Dichroweb was not possible due to the absorbance of TMAO at wavelengths lower than 205 nm, which produces a low signal-to-noise ratio, the more negative features at 210 nm and at 222 nm are indicative of a more pronounced α-helical content (Fig. 3b). Furthermore, SDSL-EPR of *Sp*UreG-MTSL indicates an important decrease of the sharp component, while the less flexible conformer reaches almost 90% at 2 M TMAO (Fig. 3a and Supplementary Table 1).

Sodium dodecyl sulfate (SDS) is often used to stabilize protein structures in IDPs^38, 39^. The titration of SDS onto the protein solution, resulted, as for TFE and TMAO, in a gain of the secondary structure composition by CD, with a maximum effect at at 1 mM SDS (Fig. 3b), a value above the critical micelle concentration (CMC) of the detergent under these buffer conditions (CMC = 0.9 mM in 20 mM TrisHCl, pH 8, 150 mM NaCl, 1 mM TCEP), measured by fluorescence^40^. A fit of the experimental CD curve indicates that the change in secondary structure mainly affects the α-helical content, which increases to 30%, while the β-strand composition decreases to 16% at 2 mM SDS. The tertiary structure of the protein also responds to the interaction with SDS micelles. In particular, at 2 mM SDS the narrower component becomes minor (17%), while the broader component becomes more flexible, with *τ*_*c*_ = 1.7 ns, to be compared with *τ*_*c*_ = 5.5 ns of the native state (Fig. 3a and Supplementary Table 1). The folding effects observed by CD and EPR are only visible when the protein interacts with SDS micelles. Performing the same experiments in a low-salt buffer (20 mM TrisHCl pH 8, 1 mM TCEP for CD; 20 mM TrisHCl pH 8, for EPR) with a CMC value higher than the concentration used for the detergent (CMC = 4 mM, in 20 mM TrisHCl pH 8, 1 mM TCEP determined by fluorescence) does not produce any significant change of the CD or the EPR. Under all the experimental conditions tested, the EPR spectrum of the free nitroxide was recorded, to confirm the absence of any effect of the used additives on the MTSL mobility (Supplementary Figure 6).

### The activity of SpUreG does not correlate with its degree of folding

**Figure 4:**
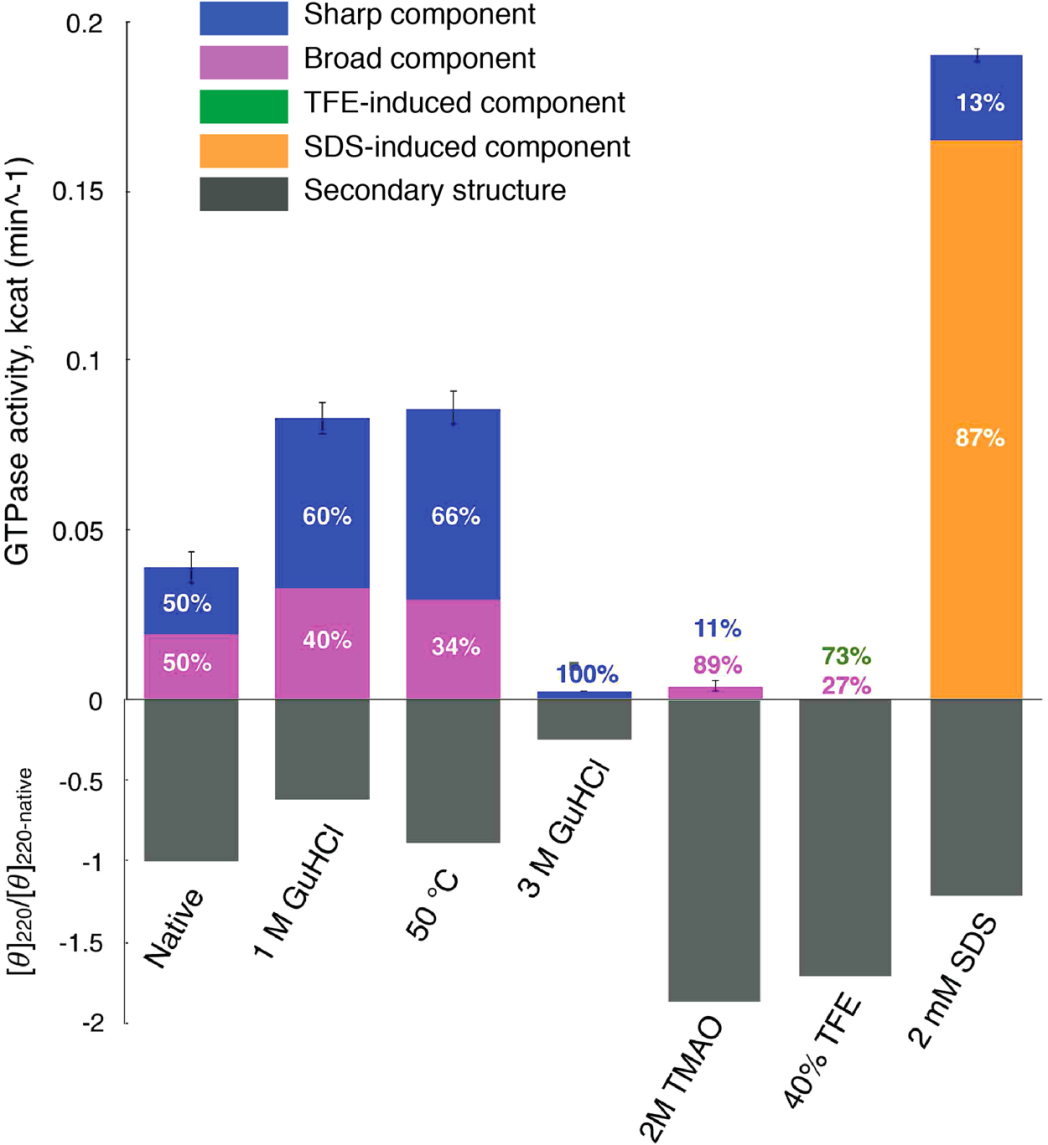
Correlation between the GTPase activity of *Sp*UreG and its structural features. The turnover number, measured with a malachite-green colorimetric assay, is reported under the addition of different additives or at different temperatures, as indicated in the figure. The error bars were calculated as the standard deviations of three replicates. The conformational distribution is plotted in terms of the relative amount of the conformations obtained from SDSL-EPR experiments, and of the content in secondary structure calculated as the ratio between the negative mean residue ellipticity, measured at 220 nm for each condition, and its absolute value measured for the native condition.

To understand how the folding and unfolding transitions, induced by the various additives and the temperature, as reported above, influence the catalytic functionality of *Sp*UreG, we measured the protein activity under the different solution conditions using a colorimetric assay^22^, and correlated the results to the structural properties observed for the protein (Fig. 4 and Supplementary Figure 2b). Under native conditions, isolated *Sp*UreG shows a slight but measurable GTPase activity, with *k*_*cat*_ = 0.039 min^−1^ ^18^. This value is lower but comparable with the one obtained for isolated NTPases of the same family, such as well-folded HypB (*k*_*cat*_ = 0.18 min^−1^) and CooC (*k*_*cat*_ = 0.15 min^−1^), involved in the biogenesis of other nickel-enzymes^41–45^, and is similar to the one determined for the intrinsically disordered GTPase TPPP/p25 (*k*_*cat*_ = 0.018 min^−1^), whose activity was also confirmed by ^31^P NMR^14^. At 50 °C, the activity of *Sp*UreG doubles up (*k*_*cat*_ = 0.086 min^−1^), indicating that the protein retains the functional activity in a conformational ensemble similar to a pre-molten globule state^23^ (Fig. 4 and Supplementary Figure 2b). As reported above, a partially denatured pre-molten globule-like conformational ensemble is also obtained at room temperature by adding 1 M GuHCl^23^. Indeed, the activity of *Sp*UreG under these conditions results at a similar level (*k*_*cat*_ = 0.083 min^−1^). These data support the idea that the protein remains functionally competent while undergoing a partial destabilization of its structure. On the other hand, the full disruption of the protein fold with temperature or high concentrations of chemical denaturants fully inactivates the enzyme: no activity was detected for the protein in the presence of 3 M GuHCl or at 80 °C, conditions in which the protein native tertiary contacts are fully abolished.

Interestingly, the transitions induced either by TFE or by TMAO toward a more rigid conformation have a dramatic effect on the protein functionality (Fig. 4 and Supplementary Figure 2b), which is totally abolished by TFE and TMAO. These results indicate that a more structured organization of the protein is not necessarily linked to the acquisition of an increased functionality and can be rather detrimental for protein activity. On the other hand, the conformational ensemble induced by SDS micelles, which features a higher α-helical content and an average mobility intermediate between the fully flexible and compact conformations, shows an increased enzymatic efficiency (*k*_*cat*_ = 0.19 min^−1^), similar to the one observed for other structured GTPases (Fig. 4 and Supplementary Figure 2b)^42, 44^. The increase in protein activity is not associated to the decrease of the backbone mobility and to an overall hardening of the protein structure: a ^1^H−^15^N HSQC NMR spectrum of the protein in the presence of 2 mM SDS does not show any major change, indicating that, under these conditions, the protein backbone remains flexible with mobility in the intermediate exchange regime (Supplementary Figure 7).

Taken together, these results suggest that while a moderate structural perturbation of the fold of *Sp*UreG does not impact on its turnover rate, either the complete disruption of the residual structure or the induction of rigid well-folded species abolishes the enzymatic activity, suggesting that some flexibility is required for enzyme functionality. The slight increase of the catalytic turnover of *Sp*UreG in the presence of low concentrations of denaturants could reflect a positive relationship between the active site flexibility and the catalytic efficiency. This observation was previously found for other enzymes, whose decrease in activity at low temperatures, attributed to the higher rigidity of the active site, was restored by the addition of low concentrations of GuHCl^46^.

Previous results of protein dynamics simulations indicated that the region of UreG containing the residues involved in enzymatic catalysis is more rigid, while other parts of the protein, especially the ones predicted to be involved in protein-protein interactions and corresponding to the region in which the paramagnetic label was situated, are more flexible and experience large conformational fluctuations^47^. The possibility of an active site structure resistant to denaturation is coherent with the ability of *Sp*UreG to retain its catalytic ability in the presence of mild denaturing conditions. The results reported here are consistent with the idea that the catalytic activity of UreG can be obtained through local contacts limited to the active site, rather than a global structural rearrangement that immobilizes the protein in a rigid conformation.

## Conclusions

Our study provides key insight into the conformational landscape of an ID enzyme, suggesting that, for *Sp*UreG, a certain degree of structural flexibility corresponds to a sweet-spot in its folding landscape, being necessary for regulating the catalytic activity through the interaction with other protein partners. The presence of slow motions is known as a universal feature for IDPs^48^ and might be also important for enzyme dynamics. These do not depend on secondary and tertiary structure propensity and are subject to kinetic barriers influenced by environmental factors, such as temperature or solution viscosity^48^. The emerging role that structural heterogeneity and conformational sampling play for an increasing number of enzymes poses new perspective for the development of new enzyme inhibitors, which is now mainly based on a rigid view of enzyme-inhibitor binding. Targeting the dynamics, instead of the structure, of enzymes is an emerging and innovative strategy to develop new drugs^49^. In addition, it supports the idea that intrinsic fold flexibility has played a role in enzyme evolution. Growing evidence suggests that archaic catalysts were molten-globule-like enzymes^50–53^, supporting a regulatory role for disorder before that subsequent protein interactions or intracellular events evolved to modulate enzymatic activity. These findings constitute a step forward to understand the role of dynamics in enzyme function and evolution, presenting also potential application for enzyme design and *in-vitro* evolution.

## Methods

### Protein purification

Purification of wild type *Sporosarcina pasteurii* UreG (*Sp*UreG) was obtained using a modification of a protocol previously reported^18^. Based on the T7 system^53^, large scale expression of *Sp*UreG was achieved in 1 L of lysogeny broth supplemented with 50 µg mL^−1^ of carbenicillin. *E. coli* BL21(DE3) cells transformed with *pET3a-SpureG* construct^54^ were grown at 37 °C with vigorous stirring. When OD_600_ reached 0.5–0.6, expression was induced by addition of isopropyl β-thiogalactopyranoside (IPTG) to a final concentration of 0.5 mM, and the temperature was decreased to 20 °C. The cells were harvested 18 h after induction by centrifugation at 6,000 rpm for 30 min at 4 °C, and resuspended in 50 ml of 50 mM Tris-HCl buffer pH 8, containing 5 mM EDTA, 2 mM DTT, 10 mM MgCl2 and 20 µg mL^−1^ DNaseI. The cells were disrupted by two passages through a French pressure cell (SLM, Aminco) at 20,000 pounds/square inch. The cell pellet was separated from the supernatant by centrifugation at 14,000 rpm for 30 min at 4 °C. The supertnatant of the lysis was loaded onto a Q-Sepharose XK 26/10 column (GE Healthcare) that had been pre-equilibrated with 2 volumes of 20 mM Tris-HCl buffer pH 8, containing 1 mM DTT and 5 mM EDTA. The column was washed using a flow rate of 3 mL min^−1^ with the starting buffer until the base line was stable. The protein was eluted from the column with a 300-ml linear gradient of NaCl (0–1 M). Fractions containing *Sp*UreG were combined and (NH_4_)_2_SO_4_ was added up to 1 M and the protein solution was centrifuged at 14,000 rpm and 4 °C for 30 minutes, in order to remove any precipitated protein. The supernatant was applied on a Phenyl Sepharose 16/10, equilibrated with 20 mM TrisHCl pH 8, containing 1 M (NH_4_)_2_SO_4_, 1 mM DTT and 2 mM EDTA. The column was washed at 2 mL min^−1^ with the starting buffer until the base line was stable. Subsequently, the protein was eluted from the column with a 250-ml linear gradient of (NH_4_)_2_SO_4_ (1–0 M). Fractions containing *Sp*UreG were combined and concentrated by using 10-kDa cut-off membrane Centricon ultra-filtration units (Millipore), to a final volume of 6 ml, and loaded onto a Superdex 75 XK 16/60 column conditioned with 20 mM TrisHCl buffer, pH 8, containing 0.15 M NaCl and 1 mM TCEP. *Sp*UreG was eluted at a flow rate of 1 mL min^-1^, and the purified protein was concentrated to 75 µM and stored at −80 °C. Protein concentration was estimated by absorption spectroscopy, using theoretical extinction coefficient ε_280_ = 11,460 M^−1^ cm^−1^), calculated from the amino acid sequence. The protein yield was 60 mg per liter of initial culture. The concentration of the protein, calculated from ε_280_ = 10,810 M^−1^ cm^−1^ ^53^, is expressed referring to the monomer.

### Circular Dichroism Spectroscopy

The CD spectra of 20 µM *Sp*UreG and *Sp*UreG-MTSL were measured at 25 °C, using a Jasco 810 spectropolarimeter flushed with N_2_, and a cuvette with 0.1-cm path length. The buffer was 20 mM TrisHCl, pH 8, containing 0.15 M NaCl and 1 mM TCEP. For the native protein, experiments were conducted in the absence and in the presence of increasing concentrations of trifluoro-ethanol (TFE, from 5% to 40%), of sodium dodecyl sulfate (SDS, from 0 mM to 2 mM) and of trimethylamine N-oxide (TMAO, from 0 M to 2 M), as reported in the Results section. The spectra were registered from 190 to 250 nm at 0.2-nm intervals. For the native protein and the protein in the presence of TMAO, only data in the 200–250 nm range are shown, due the low signal-to-noise ratio of the spectra in the 190–200 nm range, caused by buffer absorption at these wavelengths under these experimental conditions. Ten spectra were accumulated at room temperature and averaged to achieve an better signal-to-noise ratio. Spectra were normalized for protein dilution. The spectrum of the buffer was measured as a control experiment. Quantifications of protein secondary structure for TFE and SDS titrations were performed using the Dichroweb server and the CDSSTR algorithm, using reference sets 4, 7 and SP175^55^. The values obtained for secondary structure elements using the three reference sets, which presented similar statistics (NRMSD), were averaged to give the final estimation.

### Measurement of GTPase activity

GTP hydrolyzing activity was measured under different buffer conditions, as reported in the Results section, using the SensoLyte® MG Phosphate Assay Kit (AnaSpec), based on the colorimetric reaction involving malachite green reagent, molybdate and orthophosphate, as previously reported for other UreG proteins^22^. Before the analysis, the protein buffer was exchanged using PD MiniTrap G-25 desalting columns (GE Healthcare) according the manufacture’s instructions. The reaction mixture, containing 20 mM Tris-HCl, pH 8.0, 0.15 M NaCl, 5 mM MgCl2, 0.4 mM GTP, 1 mM DTT and 10 µM *Sp*UreG, in the absence or in the presence of 1 or 3 M GuHCl, 2 mM SDS, 1 or 2 M TMAO and 40% TFE, was incubated at 37 °C, at 50 °C and at 70 °C. Aliquots (100 µl) were removed at different incubation times and diluted with 400 µL of H_2_O and 100 µL of malachite green solution. Phosphate concentration was determined measuring the absorbance of the solution after 20 minutes of incubation, according to a calibration curve performed using phosphate standard solutions. Each experiment was conducted in triplicate. Phosphate concentrations measured for blank experiments, performed in parallel under the same experimental conditions, were subtracted and the resulting data were used to derive the turnover number.

### Measurement of critical micellar concentration (CMC) of SDS

SDS CMC was measured in 20 mM TrisHCl buffer pH 8, containing 1 mM TCEP and in 20 mM TrisHCl buffer pH 8, containing 150 mM NaCl and 1 mM TCEP, using the fluorescence probe N-phenyl-1-naphthylamine(NPN), according to a protocol previously reported^40^. SDS dilutions were performed from a solution stock of 250 mM in water and NPN was added to a final concentration of 1 µM. Solutions were incubated 30 mins before the analysis. Subsequently, they were analyzed with fluorescence, using an excitation wavelength of 340 nm. Emission scans were registered from 350 nm to 550 nm. The concentration of SDS in which micellization begins was calculated as the interception between the two straight lines defining the NPN emission intensity as a function of the SDS concentration^40^.

### Spin labeling of SpUreG

Protein solution containing *Sp*UreG (100 nmol) was desalted, using a PD10 desalting column (GE Healthcare), to remove the TCEP reducing agent. The buffer used was 20 mM TrisHCl pH 8, 150 mM NaCl. The spin label MTSL was then added to the protein solution in 10-fold molar excess as compared to the protein. The reaction mixture was incubated for 3.5 h in the dark under gentle stirring under a continuous flow of argon. The excess of unbound spin label was finally removed using a PD10 desalting column, using 20 mM TrisHCl pH 8, 150 mM NaCl. Labeled protein concentrations were obtained from absorbance at 280 nm, as described above.

### EPR spectroscopy

All the EPR spectra were recorded at room temperature with a spectrometer equipped with a Super High Q sensitivity resonator operating at X band (9.9 GHz). Samples were injected in a quartz capillary whose sensible volume was 40 µL.

The parameters used were the following: microwaves power = 10 mW; magnetic field modulation amplitude = 0.1 mT; field sweep = 15 mT; receiver gain = 60 dB. The labeling quantification was obtained by comparing the protein concentration with the label concentration, calculated from the double integration of the CW EPR spectrum under non-saturating conditions, and comparing it with the one measured for a standard sample. All EPR spectra were simulated using SimLabel program (a GUI of EasySpin software)^31, 32^. This program has been developed to simulate and fit CW EPR spectra, especially resulting from SDSL-EPR experiments. SimLabel allows in particular dealing with multi-components spectra often encountered in SDSL-EPR experiments. It enables determining the proportion of the different components and for each of them describing all the parameters: magnetic parameters (g- and A-tensors) and the dynamic parameter τ_c_ corresponding to the rotational correlation time.

### MALDI-ToF MS Spectrometry

*Determination of Global Mass of unlabeled and labeled* UreG. Mass analysis (MALDI-ToF) was performed to confirm the labeling (theoretical mass increment resulting for one MTSL grafted on the protein is of 184.2 Da).

Samples of ~70 pmol of UreG (unlabeled) and UreG^MTSL^ (labeled) were prepared by dilution in 10 µL of 0,1% of trifluoro-acetic acid (TFA) in water (v/v) before being spotted onto a MALDI target plate (1 µL). A saturated solution of sinapinic acid matrix (1 µL) in 40% acetonitrile/water, 0,1% TFA (v/v) was added. The global mass was measured on a MALDI-ToF mass spectrometer Microflex II from Bruker Daltonics in the range of 2000 to 55000 Da in a positive linear mode. External mass calibration was performed using the signals of trypsinogen and protein A from the Protein standard II (Bruker Daltonics). The error on the measurement is of + /− 5 Da.

### NMR Spectroscopy

NMR spectra were acquired at 298 K on a Bruker DRX 700 MHz spectrometer operating at the proton frequencies of 700.13 MHz (16.44 T) on uniformly ^15^N- enriched samples of ca. 0.5 mM UreG in 20 mM TrisHCl buffer at pH 8, containing 150 mM NaCl, 1 mM TCEP, in the absence and in the presence of 2 mM SDS. The spectrometer was equipped with a triple-resonance 5-mm TCI cryo-probe with pulse field gradients along the z-axis. The spectra were acquired using 2048 × 128 number of complex points and the hsqcfpf3gpphwg Bruker library pulse sequence.

## Electronic supplementary material

Supplementary information
