# Supplementary material for: The relationship between folding and activity in UreG, an intrinsically disordered enzyme
Source: Sci Rep. 2017 Jul 20;7:5977. doi: 10.1038/s41598-017-06330-9 (PMC5519622; doi:10.1038/s41598-017-06330-9)
Supplement: Supplementary file 1 — Supplementary information [file 41598_2017_6330_MOESM1_ESM.pdf]

The relationship between folding and activity in UreG, an intrinsically disordered enzyme

Marta Palombo,[1] Alessio Bonucci,[2] Emilien Etienne,[2] Stefano Ciurli[1], Vladimir Uversky,[3] Bruno Guigliarelli, [2] Valérie Belle,[2] Elisabetta Mileo[2,*], Barbara Zambelli[1,*]

[1]Laboratory of Bioinorganic Chemistry, Department of Pharmacy and Biotechnology, University of Bologna, Viale G. Fanin 40, 40127 Bologna (Italy)

[2] Aix-Marseille Univ, CNRS, IMM (FR 3479), BIP (UMR 7281), 31 chemin Joseph Aiguier 13402 Marseille (France)

[3]Department of Molecular Medicine, University of South Florida, 12901 Bruce B. Downs Blvd., MDC07, Tampa (USA)

*barbara.zambelli@unibo.it; emileo@imm.cnrs.fr

Supplementary Information

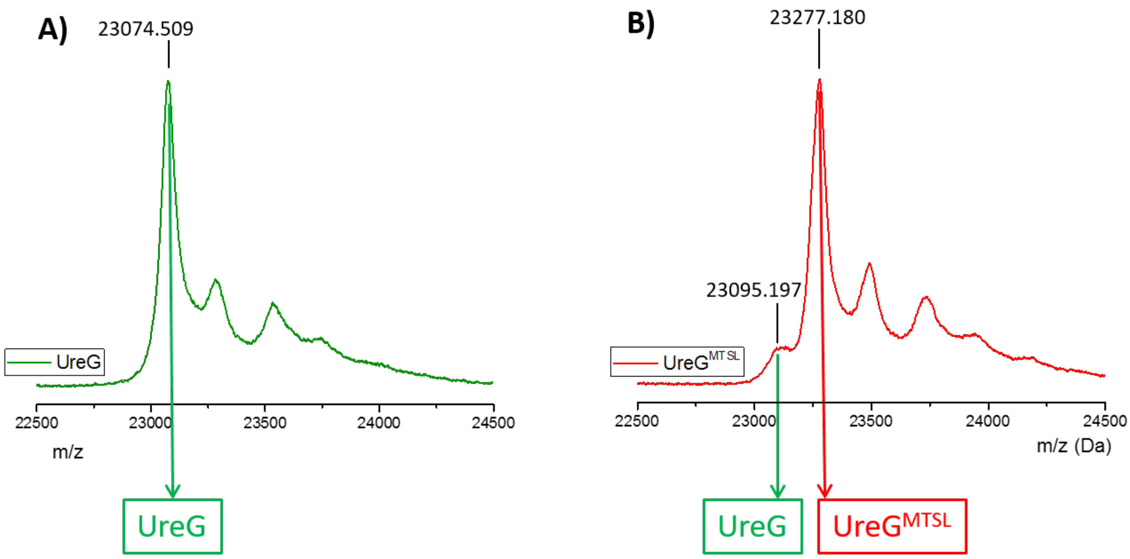

Supplementary Figure 1: MALDI-ToF analysis of native *Sp*UreG *vs*. MTSL-labeled *Sp*UreG: A) Unlabeled and B) MTSL-labeled *Sp*UreG. The theoretical mass increment due to the labeling of one MTSL is 184.20 Da The experimental value of the mass increment is 181,98 Da. According to the error of the measurement (+/- 5 Da), the experience confirms the labeling of *Sp*UreG with a single MTSL probe.

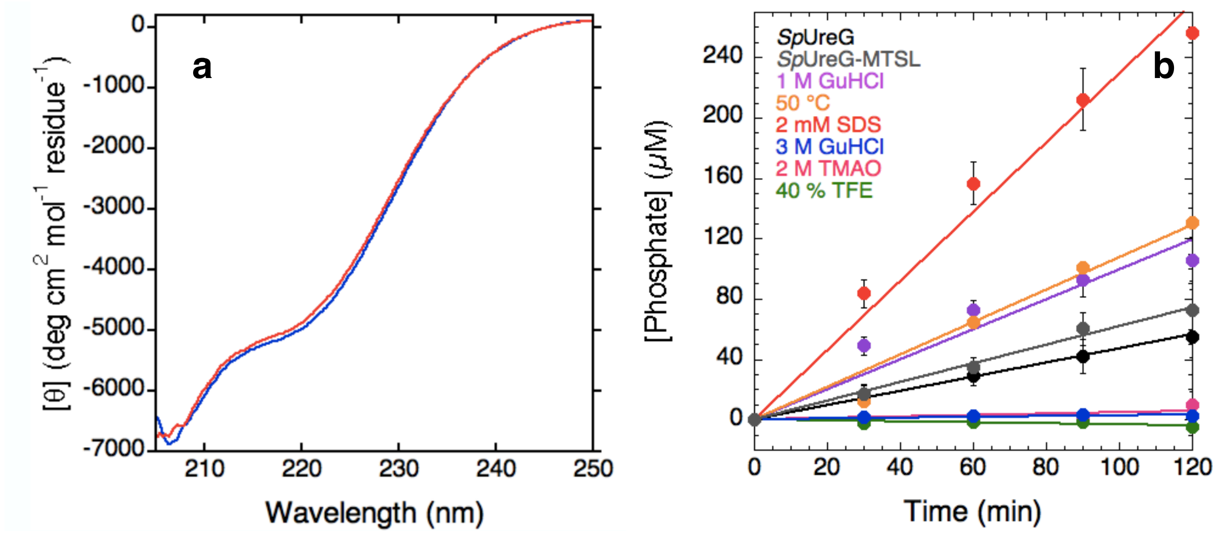

Supplementary Figure 2 – a. Circular dichroism of the native *Sp*UreG (20 μM) in 20 mM TrisHCl pH 8, 150 mM NaCl, 1 mM TCEP (*blue*)[1] and of *Sp*UreG with the MTSL nitroxide bound to Cys[68], under the same experimental conditions. b Time course of GTP hydrolysis of *Sp*UreG (circles) under different experimental conditions as indicated in the figure. Solid lines indicate the fit of the data using a linear regression.

A)

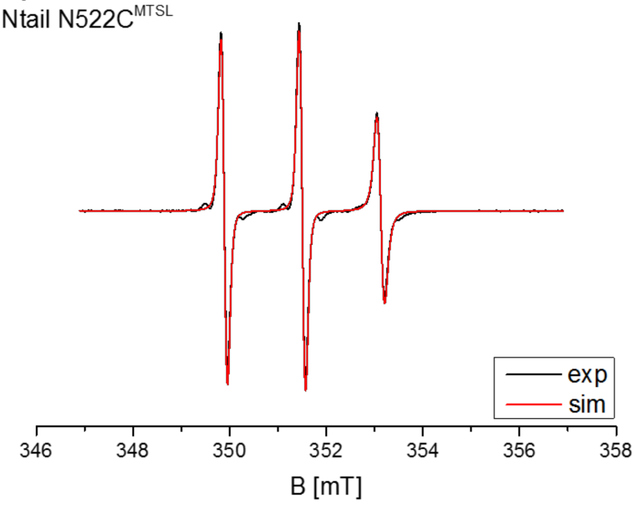

B)

g_x	2.00888
g_y	2.00610
g_z	2.00220
a_x, a_y(mT)	0.50
a_z(mT)	3.87
a_{iso} (mT)	1.62
LW (mT)	G: 0.13 L: 0.02
τ_c (ns)	0.18
%	100

Supplementary Figure 3 –A) Experimental EPR spectrum of Ntail^{MTSL} (black line) labeled at position 522 and its simulation (red line) with SimLabel program[2] (a GUI of EasySpin)[3]. B) Parameters resulting from the simulation.

A) UreG^{MTSL} native 62µM

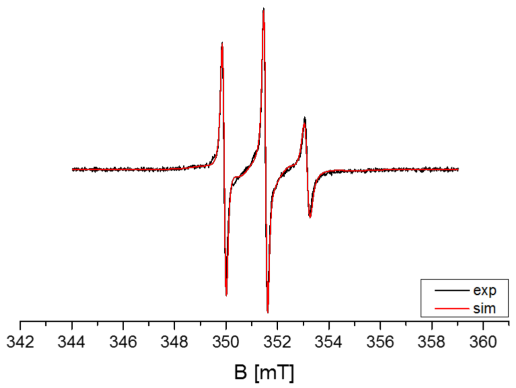

	« sharp » component	« broad » component
g_x	2.00792	2.00606
g_y, g_z	2.00610, 2.00220	2.00610, 2.00220
a_x, a_y(mT)	0.51	0.64
a_z(mT)	3.84	3.69
a_{iso} (mT)	1.62	1.66
LW (mT)	G: 0.12 L: 0.02	G: 0.12 L: 0.02
τ_c (ns)	0.43	5.5
%	50	50

B) UreG^{MTSL} native 264µM

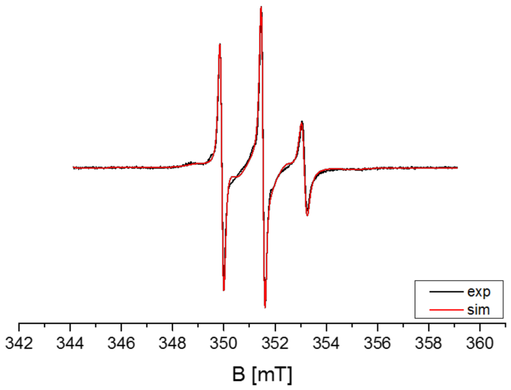

	« sharp » component	« broad » component
g_x	2.00792	2.00606
g_y, g_z	2.00610, 2.00220	2.00610, 2.00220
a_x, a_y(mT)	0.51	0.64
a_z(mT)	3.84	3.69
a_{iso} (mT)	1.62	1.66
LW (mT)	G: 0.12 L: 0.02	G: 0.12 L: 0.02
τ_c (ns)	0.43	5.5
**%**	**42**	**58**

Supplementary Figure 4: Experimental EPR spectra (black line) and their simulation (red line) of *Sp*UreG-MTSL 62µM (A) and 264 µM (B) in 20 mM TrisHCl buffer pH 8, 150 mM NaCl at 25 °C. A table summarizing the results from simulations is reported below each spectrum. $g_{x, y, z}$ and $a_{x, y, z}$ correspond to g-tensor and hyperfine-tensor values. a_{iso} is the mean value of a_x, a_y and a_z. LW describes the line width resulting from a convolution between a Gaussian (G) and a Lorentzian (L) function. τ_c is the rotational correlation time and % represents the proportion of the component.

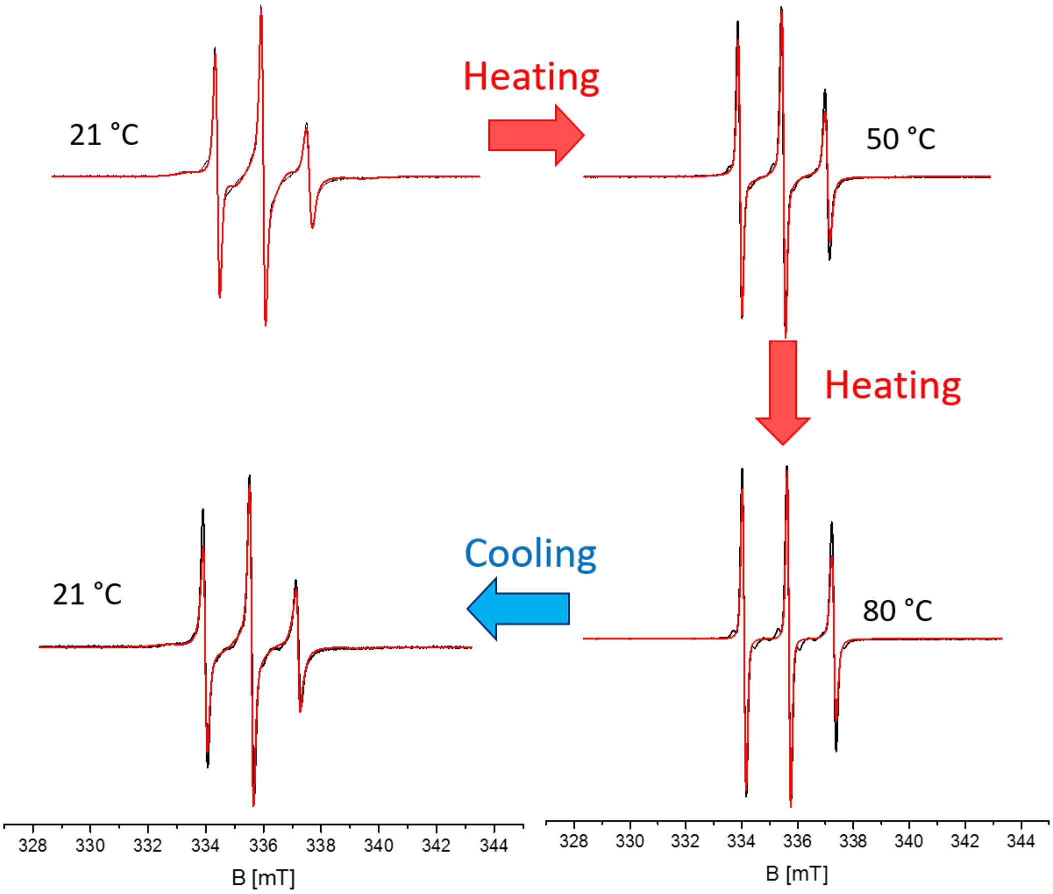

Supplementary Figure 5: Experimental EPR spectra (black line) and simulations (red line) of 80µM *Sp*UreG-MTSL in 20 mM TrisHCl buffer pH 8, 150 mM NaCl at 21 °C, 50 °C, 80 °C and 21 °C after temperature increase and subsequent decrease.

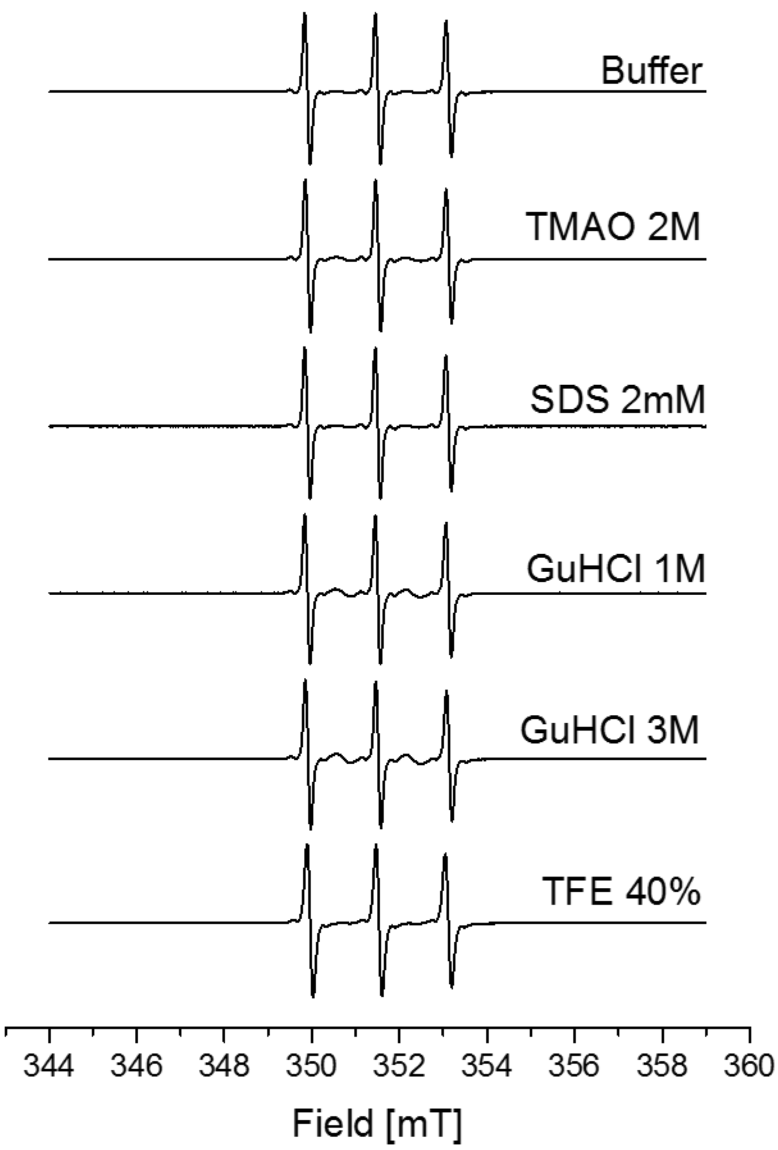

Supplementary Figure 6: Experimental EPR spectra of the spin label MTSL (130µM, not attached to the protein) in 20 mM TrisHCl buffer pH 8, 150 mM NaCl at room temperature and in the same experimental conditions described in the text for *Sp*UreG-MTSL.

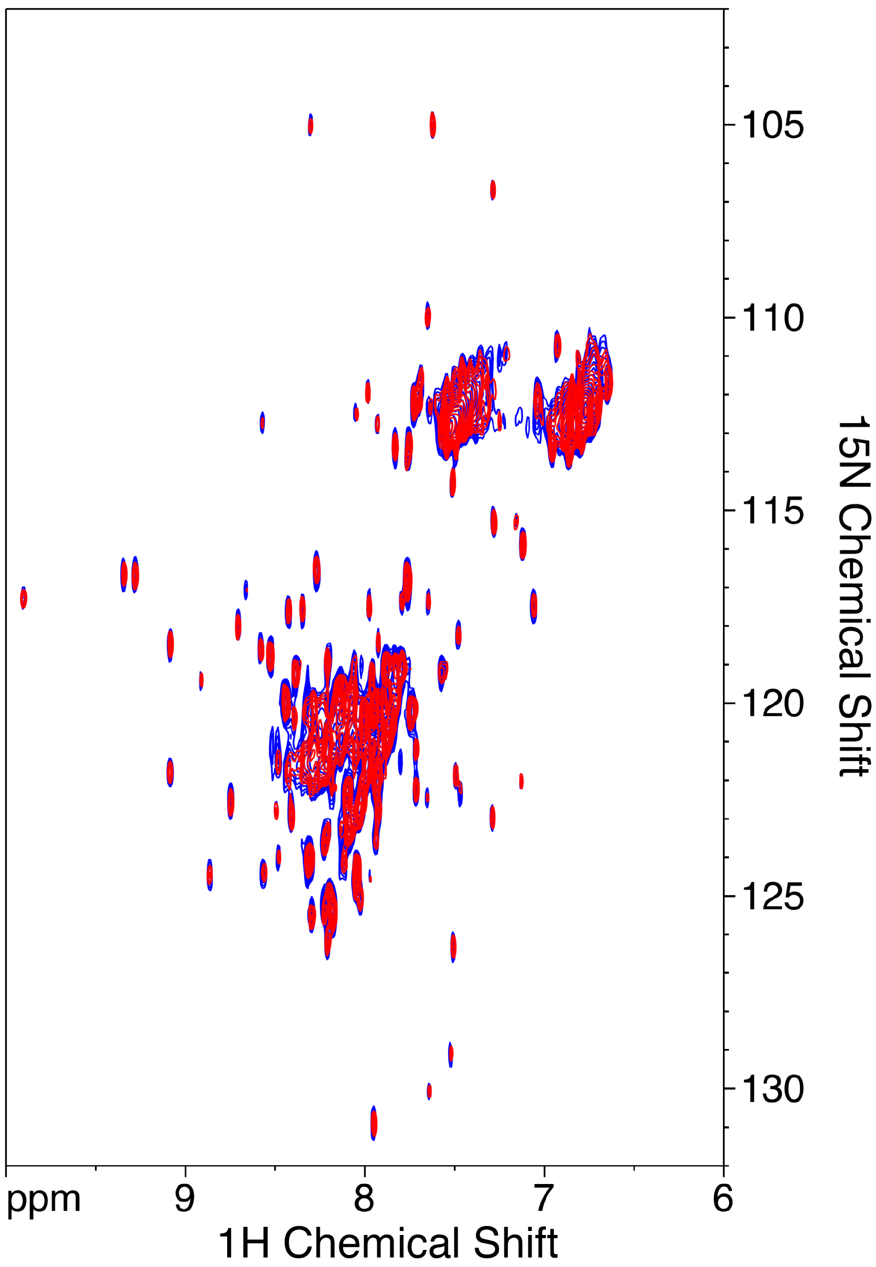

Supplementary Figure 7: $^{1}H,^{15}N$ HSQC 700 MHz NMR spectrum of 0.5 mM *Sp*UreG in 20 mM TrisHCl pH 8, 150 mM NaCl, 1 mM TCEP, in the absence (red) and in the presence (blue) of 2 mM SDS.

Supplementary Table 1. SimLabel (a GUI of EasySpin) was used to simulate EPR spectral shapes. The principal values of the g-tensor are: g_x in the range of [2.00588, 2.00810], g_y=2.00610 and g_z=2.00220. The ^{14}N hyperfine tensor is considered axial and parallel to g-tensor in the range of [0.50, 0.64]mT and A_z in the range of [3.69, 3.99]mT.

	sharp		broad	
	τ_c (ns)	%	τ_c (ns)	%
**Native**	0.43	50	5.5	50
**1 M GuHCl**	0.43	60	5.5	40
**3 M GuHCl**	0.43	100	/	/
**50 °C**	0.31	66	4.0	34
**1M TMAO**	0.49	23	5.5	77
**2 M TMAO**	0.49	11	5.5	89
**40% TFE**	0.73	73	5.5	27
**2 mM SDS**	0.44	13	1.7	87

References

1 Zambelli, B. et al. UreG, a chaperone in the urease assembly process, is an intrinsically unstructured GTPase that specifically binds Zn2+. J. Biol. Chem. 280, 4684-4695, doi:10.1074/jbc.M408483200 (2005).

2 Etienne, E., Le Breton, N., Martinho, M., Mileo, E. & Belle, V. SimLabel: a graphical user interface to simulate continuous wave EPR spectra from Site-Directed Spin Labeling experiments. Magn. Reson. Chem., doi:10.1002/mrc.4578 (2017).

3 Stoll, S. & Schweiger, A. EasySpin, a comprehensive software package for spectral simulation and analysis in EPR. J. Magn. Reson. 178, 42-55, doi:10.1016/j.jmr.2005.08.013 (2006).
